# Formation of a morphine-conditioned place preference does not change the size of evoked potentials in the ventral hippocampus–nucleus accumbens projection

**DOI:** 10.1038/s41598-019-41568-5

**Published:** 2019-03-26

**Authors:** D. Y. Sakae, S. J. Martin

**Affiliations:** 1 Division of Systems Medicine, University of Dundee, Ninewells Hospital and Medical School, Dundee, DD1 9SY UK; 2 Present Address: Robarts Research Institute, University of Western Ontario, 1151 Richmond Street North, London, ON N6A 5B7 Canada

## Abstract

In opioid addiction, cues and contexts associated with drug reward can be powerful triggers for drug craving and relapse. The synapses linking ventral hippocampal outputs to medium spiny neurons of the accumbens may be key sites for the formation and storage of associations between place or context and reward, both drug-related and natural. To assess this, we implanted rats with electrodes in the accumbens shell to record synaptic potentials evoked by electrical stimulation of the ventral hippocampus, as well as continuous local-field-potential activity. Rats then underwent morphine-induced (10 mg/kg) conditioned-place-preference training, followed by extinction. Morphine caused an acute increase in the slope and amplitude of accumbens evoked responses, but no long-term changes were evident after conditioning or extinction of the place preference, suggesting that the formation of this type of memory does not lead to a net change in synaptic strength in the ventral hippocampal output to the accumbens. However, analysis of the local field potential revealed a marked sensitization of theta- and high-gamma-frequency activity with repeated morphine administration. This phenomenon may be linked to the behavioral changes—such as psychomotor sensitization and the development of drug craving—that are associated with chronic use of addictive drugs.

## Introduction

The global health and social costs of opioid addiction are substantial and growing^1^. Learned associations between drug-related cues and drug reward can lead to cravings that are powerful drivers of continued opioid use, or relapse in formerly abstinent users^2–4^. One structure that is involved in the processing of certain types of drug-associated cue is the hippocampal formation^2^. The hippocampus exhibits anatomical and functional heterogeneities along its longitudinal axis, with the dorsal part of the structure closely associated with spatial learning and memory, and the ventral region implicated in motivation, emotion, and anxiety^5,6^. The ventral hippocampus (VH) nonetheless plays a significant role in spatial memory^7–11^, as well as the processing of contextual information^12^. The VH also provides a key link between hippocampal place information and the emotional, motivational, and executive systems of the brain^13–15^.

Consistent with this role, there is a prominent glutamatergic projection from area CA1 and the subiculum of the VH to the medium spiny neurons (MSNs) of the shell region of the nucleus accumbens (NAcS)^16–19^. Electrical stimulation of ventral CA1 or subiculum, or downstream efferents in the fimbria/fornix, elicits characteristic evoked field potentials (EFPs) in the NAc^20–25^. The VH-NAcS pathway may convey spatial and contextual information to the NAc, which then links this information to the presence or absence of reward^26^. This projection is therefore well placed to play a central role in both the formation of associations between environmental context and reward, and in context-induced relapse.

A commonly used experimental model for the association of context and reward is conditioned place preference (CPP), in which drug administration—e.g. cocaine or morphine—is repeatedly paired with a specific place or context, leading to a conditioned preference for this location, even in the absence of reward. In support of the idea that the VH-NAcS plays a role in context–reward learning, disconnection of the ventral subiculum and NAcS, prevents the acquisition of CPP^27^ as does optogenetic silencing of this projection^28^.

Consistent with its potential role in memory formation, the VH-NAc projection is plastic, exhibiting increases in synaptic strength in response to high-frequency electrical stimulation (e.g.^23,28^); these changes can be blocked by the application of an NMDA-receptor antagonist^28–30^. There is substantial indirect evidence from *ex vivo* experiments involving brain slices collected after training that cocaine-induced CPP causes changes in synaptic strength at excitatory synapses of NAc MSNs^19,31–33^, and changes in the coupling of hippocampal and MSN unit activity^34^. There is less evidence concerning morphine CPP, but a selective potentiation of excitatory inputs to MSNs expressing dopamine D1 receptors (D1Rs) has recently been reported after repeated morphine administration^35^. However, synaptic changes have never been directly recorded in the NAc of intact, behaving animals during the formation of a context-reward association.

We therefore implanted rats with chronic electrodes for the recording of field potentials in the NAcS evoked by stimulation of ventral CA1/subiculum. The local field potential (LFP) was also monitored via the same electrode throughout the experiment. Animals were trained in a conditioned-place-preference (CPP) task in which morphine injection was associated with one of two chambers with distinctive visual and tactile features. Both NAcS EFP and LFP data were monitored during the acquisition and extinction of CPP.

## Results

### Properties of VH-NAcS synaptic transmission

**Figure 1:**
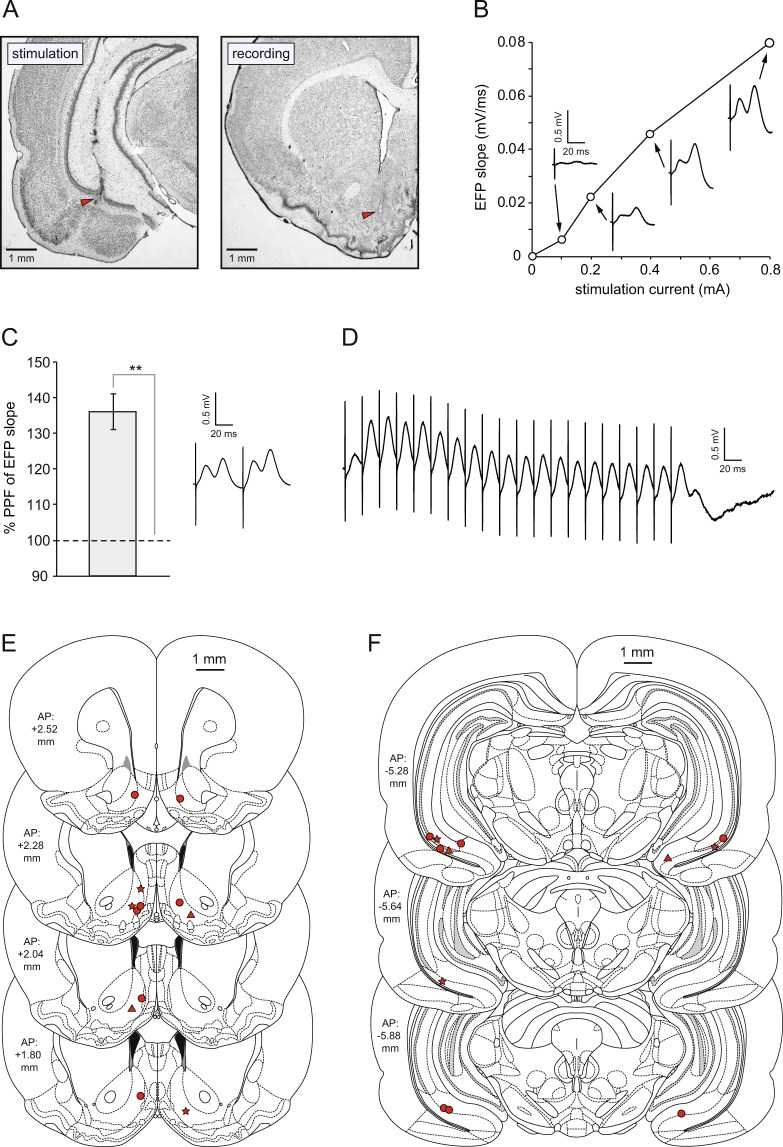
Electrode placements and properties of the ventral hippocampus – nucleus accumbens projection. (**A**) Examples of photomicrographs of histological sections showing stimulation and recording sites (red arrows). (**B**) Example of an input-output curve indicating the slope of the rising phase of the first positive peak of the evoked field potential (EFP) as a function of stimulation intensity. (**C**) Example of paired-pulse facilitation from a single rat, at an inter-stimulus interval of 50 ms. A representative pair of EFPs is shown. (**D**) EPSs elicited by stimulation at 50 Hz. (**E**) Locations of recording electrodes and (**F**) stimulating electrodes, based on histological sections [⚫ = main experiment (n = 7); ★ = morphine time-course experiment (n = 3); ▲ = preliminary experiments (paired-pulse facilitation, I/O curve, and 50 Hz; n = 2). Atlas sections adapted from Paxinos and Watson (2004).

Consistent with previous reports (e.g.^25^), stimulation of the ventral CA1/subiculum (Fig. 1A) resulted in a characteristic multi-component evoked field potential (EFP) in the NAcS with a positive peak at approximately 12 ms, and a second, longer-latency positive component. Examples elicited by a range of stimulation intensities are shown in Fig. 1B; the slope of the rising phase of the first positive peak, in this case measured between 6–7.5 ms after stimulation, is plotted as a function of intensity; each data point is the average of 4 values from the same animal. Paired-pulse stimulation at an interval of 50 ms resulted in a marked facilitation of the slope of the initial positive component of the second pulse relative to the first (Fig. 1C); the bar graph shows the mean percentage facilitation of 9 successive paired pulses in the same rat [t(8) = 7.37; p < 0.0005]. Figure 1D shows a train of responses to stimulation at 50 Hz. The first positive peak of the EFP reliably followed each stimulation pulse with a constant onset latency. Histological analysis of post-mortem brain sections revealed that, in all rats used in the study, recording electrodes were located in the NAcS (Fig. 1E), and stimulating electrodes were located in ventral CA1 or subiculum (Fig. 1F).

### Behavioral testing

**Figure 2:**
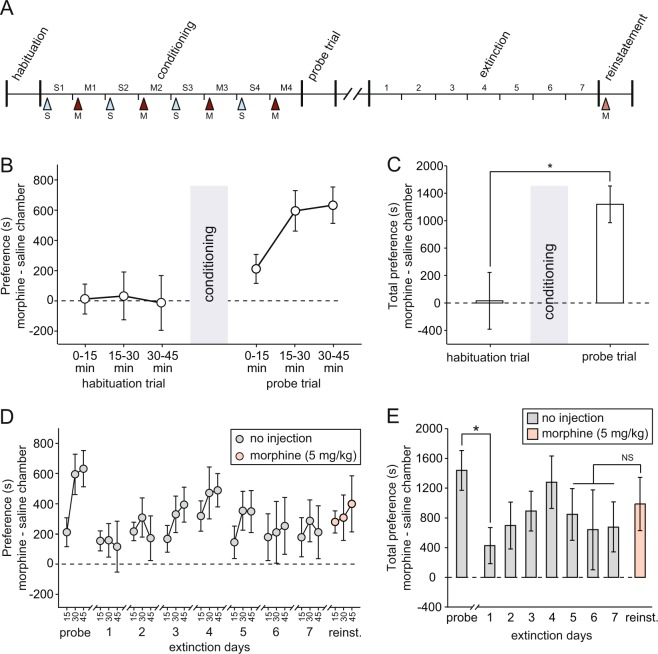
Place preference data (n = 7 throughout). (**A**) Time-line of experiment (S = saline injection; M = morphine injection; S1 = saline day 1; M1 = morphine day 1, etc.). (**B**) Place preference (expressed as the difference between time spent in the saline versus morphine-associated chambers) during the habituation and probe trials, i.e. before and after conditioning. Each trial is divided into three 15-min time bins. (**C**) Mean place preference before and after conditioning across the whole 45-min of each trial. (**D**) Place preference during the probe, extinction, and reinstatement trials. (**E**) For the same trials as in D, mean place preference across the whole 45 min.

A time-line of the different phases of CPP testing is shown in Fig. 2A. Throughout the analysis, we have divided each testing session into 15-min time windows to highlight changes within a session, as well as across days. We used the difference between time spent in the morphine versus saline-associated chamber as a measure of place preference throughout. A 2-way repeated measures ANOVA in which trial and time window were entered as within-subjects factors revealed a significant increase in preference between habituation and probe trials [Fig. 2B,C; F(1,6) = 7.94; p = 0.03], but no interaction between trial and time-interval [F(2,12) = 3.17; p = 0.08]. During the habituation trial, mean preference for the future morphine chamber did not differ from chance [Fig. 2C, left-hand bar; t(6) = 0.08; p = 0.94; one-sample t-test]; however, a significantly above-chance preference for the morphine-associated side was evident in the probe trial conducted after conditioning [Fig. 2C, right-hand bar; t(6) = 5.39; p = 0.004; one-sample t-test with Benjamini-Hochberg correction for multiple comparisons].

Preference for the morphine chamber during extinction is shown in Fig. 2D & E. There was significant forgetting between the probe trial and extinction day 1 [t(6) = 3.63; p = 0.011]. However, an ANOVA of the 7 extinction days revealed no further change in preference [F(6,36) = 0.78; p = 0.59]; mean preference over the final 3 extinction days did not differ significantly from chance [t(6) = 2.30; p = 0.061]. A priming dose of morphine caused a non-significant increase in preference for the morphine-associated chamber in the reinstatement trial, relative to the mean of the final 3 extinction trials [Fig. 2D & E; right-hand-side; t(6) = 0.78; p = 0.46]. However, an analysis of the mean difference in distance traveled in the formerly morphine and saline-associated chambers (i.e. ‘distance preference’ rather than ‘time preference’) revealed a significant increase in this measure after reinstatement [mean of final 3 extinction days: 5.17 ± 4.36 m; reinstatement trial: 48.92 ± 17.92 m; t(6) = 2.72; p = 0.035], suggesting that active exploration of the morphine-associated chamber was increased by the priming dose of morphine, but not total time spent within this chamber.

**Figure 3:**
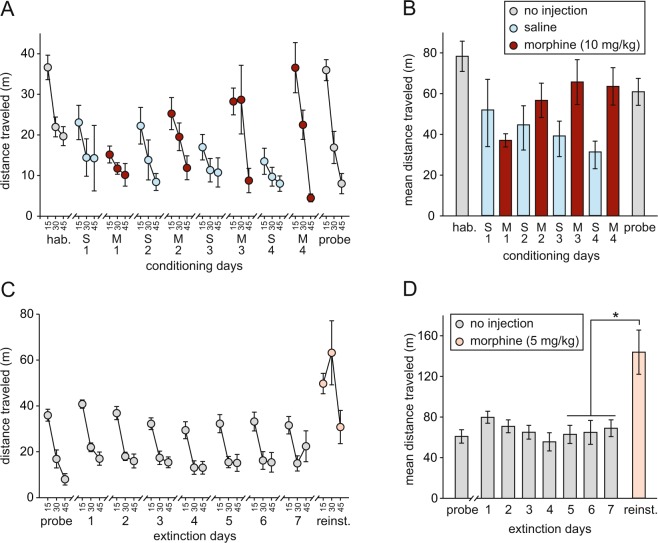
Morphine-induced locomotor sensitization (n = 7 throughout). (**A**) Distance traveled during habituation (hab.), conditioning, and probe trials, divided into 15-min time bins (S1 = saline day 1; M1 = morphine day 1, etc.). (**B**) Total distance traveled during the whole 45 min of each of the trials plotted in (**A**). (**C**) Distance traveled during probe, extinction, and reinstatement (reinst.) trials, divided into 15-min time bins. (**D**) Total distance traveled during the whole 45 min of each of the trials plotted in (**C**).

Figure 3A,B show total distance traveled—i.e. locomotor activity—during habituation and conditioning. Activity declined significantly across the three 15-min time bins of the habituation trial [F(2,12) = 79.9; p < 0.0005; Fig. 3A, left-hand side], indicating within-session habituation to the apparatus, a pattern that was repeated across subsequent trials. But whereas only modest changes in activity were evident across saline days, there was a progressive sensitization of the locomotor response to morphine with successive injections. The very first injection of morphine caused a slight reduction in activity relative to the preceding saline day, but subsequent exposures resulted in progressively increasing locomotor activity during the first 30 min of each trial, relative to activity levels on the intervening days. However, activity levels during the last 15 min of each session fell slightly with successive injections.

An ANOVA in which drug (morphine versus saline), testing day (saline days 1–4 & morphine days 1–4; S1–S4 and M1-M4 in Fig. 3A), and time (i.e. within-session 15-min time-window) were all entered as within subjects factors revealed a significant triple interaction of drug × day × time [F(2.25,36) = 4.05; p = 0.038; Greenhouse-Geisser correction]. Analysis of the first 15-min time-window only, the point at which morphine-induced locomotion was typically largest, revealed a highly significant drug × day interaction [F(3,18) = 7.35; p = 0.002], reflecting the widening difference between saline and drug days as conditioning progressed: activity increased across morphine days, but decreased across saline days. Comparing the final saline and morphine days only (S4 and M4 in Fig. 3A), there was a significant overall difference between drug and saline conditions [F(1,6) = 6.25; p = 0.047], and a drug x time interaction [F(2,12) = 14.6; p = 0.001]; pairwise comparisons (paired-sample t-tests with Benjamini-Hochberg corrections for multiple comparisons) revealed that morphine caused an increase in activity in the first and second 15-min windows [t(6) = 2.99; p = 0.034 and t(6) = 2.94; p = 0.034, respectively], but a decrease in activity in the final 15 min [t(6) = 2.74; p = 0.034].

The high locomotor activity levels evident during the probe trial (Fig. 3A,B; right-hand side) are likely to reflect the fact that, like the habituation trial, both chambers were available for exploration, rather than the single chamber experienced throughout the conditioning phase. These activity levels were maintained throughout the extinction phase in the absence of drug administration (Fig. 3C,D). There was no significant overall change in activity over the probe trial and 7 subsequent extinction days [F(7,42) = 1.34; p = 0.26]. However, there was an increase in activity on the reinstatement trial after a priming dose of morphine. The increase in activity was significant between the mean of the final 3 days of extinction and the mean of the reinstatement trial [t(6) = 3.02; p = 0.023; paired-sample t-test].

### Changes in evoked field potentials (EFPs)

**Figure 4:**
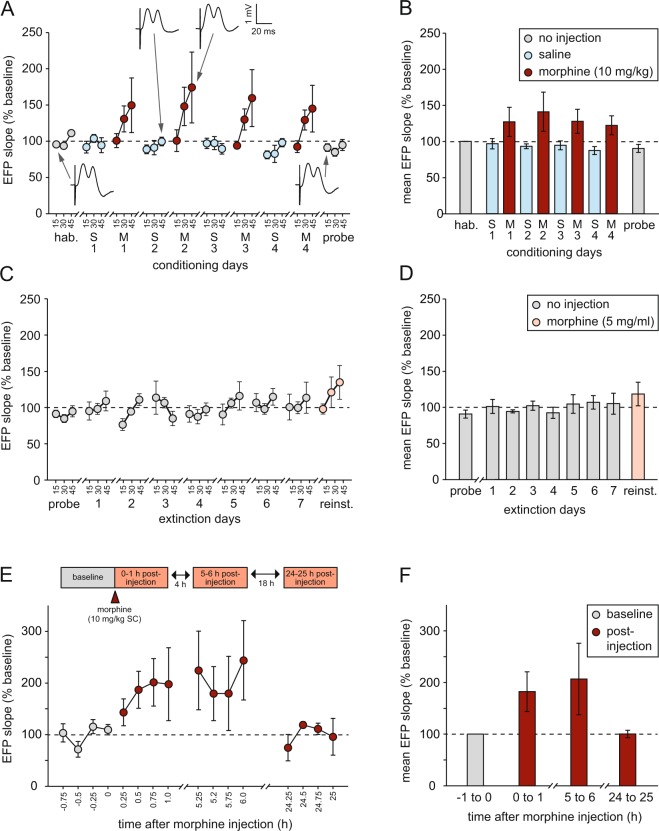
NAcS evoked field potential (EFP) data (n = 7 throughout). (**A**) EFP slope during habituation (hab.), conditioning, and probe trials, divided into 15-min time bins, and normalized to the mean value during habituation (100%) (S1 = saline day 1; M1 = morphine day 1, etc.). Representative examples are shown of EFPs recorded from the same rat during habituation, saline, morphine, and probe trials. (**B**) Mean normalized EFP slope during the whole 45 min of each of the trials plotted in (**A**). (**C**) EFP slope during probe, extinction, and reinstatement (reinst.) trials, divided into 15-min time bins, and normalized to the mean value during habituation (100%). (**D**) Mean normalized EFP slope during the whole 45 min of each of the trials plotted in (**C**). (**E**) Time course of the increase in EFP slope following morphine administration in a separate group of animals (n = 3), normalized to the mean value over the 1-h period before injection (n = 3). (**F**) Summary of the data as shown in (**E**), plotted as mean normalized hourly EFP slope values before and after morphine injection.

Figure 4A,B show the slope of the first positive component of the NAcS EFP evoked by VH stimulation before, during, and after conditioning. All values recorded within each 15-min time window were averaged and normalized to the mean value during the habituation session (designated 100%). A comparison of mean EFP slope values during habituation and probe trials—procedurally identical sessions—did not reveal a significant learning-related change [t(6) = 1.73; p = 0.14; paired-sample t-test]. Examples of EFPs recorded from a single rat during the first 15 min of each of each trial are shown. To assess any influence of the morphine- or saline-associated chamber on the size of the EFP during the probe trial, we used Any-maze tracking data to segregate EFPs according to the chamber in which the rat was located at the time of VH stimulation. This analysis focused only on the first half of the trial only (0–22.5 min), because 4/7 rats did not explore the saline-associated chamber at all during the second half of the trial. There was no difference between saline and morphine chambers in mean EFP slope over this period [saline: 85.3 ± 11.8%; morphine: 85.0 ± 5.87%; t(6) = 0.03; p = 0.98; paired-sample t-test].

During conditioning trials, little change was evident within and between saline days, but morphine caused a gradual rise in EFP slope throughout each session, although values always returned to baseline by the start of the subsequent saline session. An analysis of the mean EFP slope during the final 15 min of each conditioning session, revealed a significant increase on morphine relative to saline days [z = 2.37; p = 0.018; Wilcoxon signed-ranks test; non-parametric test applied owing to marked heterogeneity of variance between drug conditions and deviation from normality in the morphine data]. Slope values during the last 15 min did not change across days in the saline or morphine conditions alone [χ^2^ = 1.80; df = 3; p = 0.62 in both cases; Friedman tests], indicating that there was no sensitization of the effect of morphine on EFP magnitude.

No overall changes in EFP slope were seen across the probe trial and successive extinction days [Fig. 4C,D; F(7,42) = 0.78; p = 0.61]. Injection of a priming dose of morphine revealed a small increase in the EFP slope; the mean EFP slope recorded during the final 15 min of the reinstatement trial was significantly higher than the mean of the corresponding time-point of the preceding 3 extinction days [z = 2.03; p = 0.043; Wilcoxon signed-ranks test]. A study of the time-course of morphine-induced EFP changes in a separate group of rats (Fig. 4E,F) revealed a plateau within about 45 min of injection, and an increase that was maintained for at least 6 h. However, the effect returned to baseline within 24 h. A statistical analysis was not conducted owing to the small number of animals (n = 3), but all rats showed a morphine-induced increase in EFP slope.

### Changes in LFP activity

**Figure 5:**
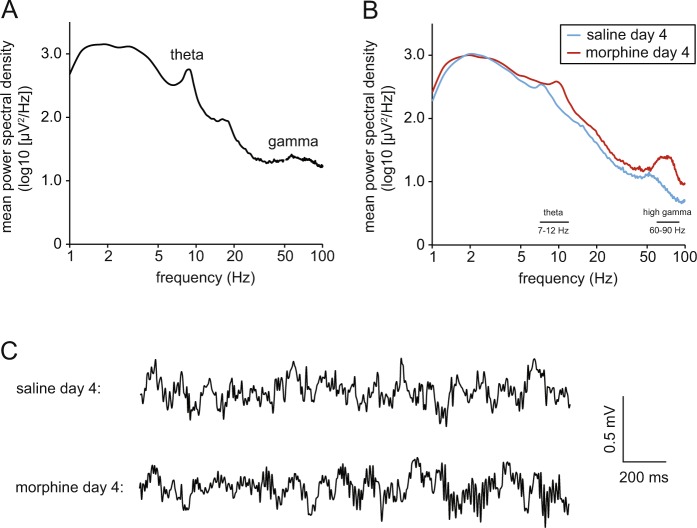
LFP power spectra. (**A**) Log-log plot of mean power spectral density as a function of frequency during the 45-min habituation trial for a single rat. (**B**) Mean power spectral density for the same rat during the final saline and morphine conditioning trials. Note the increase in theta and high gamma power in the latter. (**C**) Examples of 2-s periods of LFP activity recorded from the same rat during the final saline and morphine conditioning trials.

Figure 5A shows an example of the mean power spectral density in the NAcS as a function of frequency during the habituation trial. There is a pronounced theta-frequency peak, and a modest gamma peak at around 60 Hz. After the final morphine injection, there was an increase in the amplitude, relative to the preceding saline day, of theta activity, and high-frequency gamma (60–90 Hz), but only a modest change in low-frequency gamma (Fig. 5B). For this reason, we focus primarily on theta and high gamma in subsequent analyses. Figure 5C shows examples of LFP activity recorded during the final saline and morphine days of the conditioning phase, showing the increase in gamma activity in the latter.

**Figure 6:**
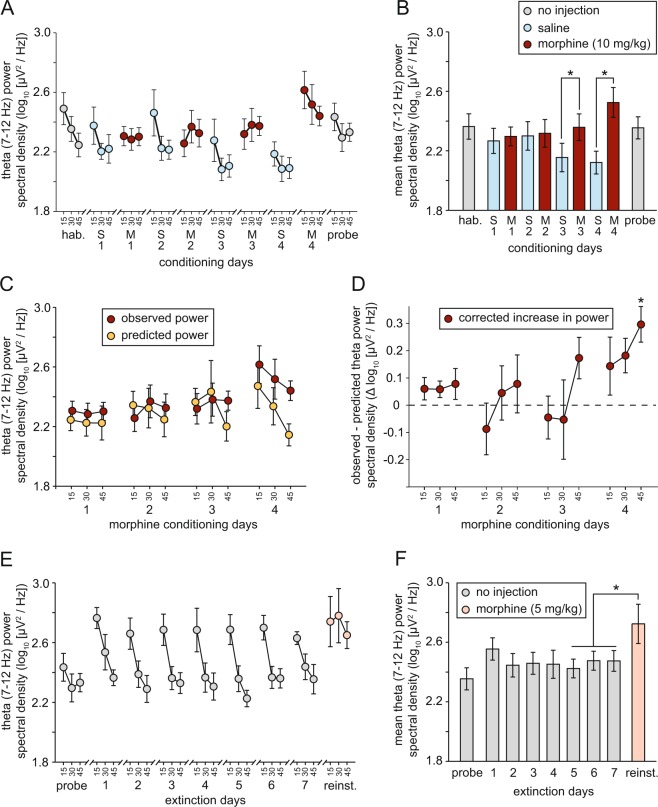
Morphine-induced sensitization of NAcS theta activity (n = 7 throughout). (**A**) Theta power spectral density during habituation (hab.), conditioning, and probe trials, divided into 15-min time bins (S1 = saline day 1; M1 = morphine day 1, etc.). (**B**) Mean theta power spectral density during the whole 45 min of each of the trials plotted in A [*p < 0.05; paired-sample t-tests with Benjamini-Hochberg correction for multiple comparisons]. (**C**) Observed power spectral density (divided into 15-min time bins) during morphine conditioning days, versus power predicted based on the positive relationship between locomotor activity and theta power; see Fig. S1A. (**D**) Morphine-induced increase in theta power corrected for locomotor activity [*p < 0.05; one-sample t-test with Benjamini-Hochberg correction for multiple comparisons]. (**E**) Theta power spectral density during probe, extinction, and reinstatement (reinst.) trials, divided into 15-min time bins. (**F**) Mean theta power spectral density during the whole 45 min of each of the trials plotted in (**E**) [*p < 0.05; paired-sample t-test].

**Figure 7:**
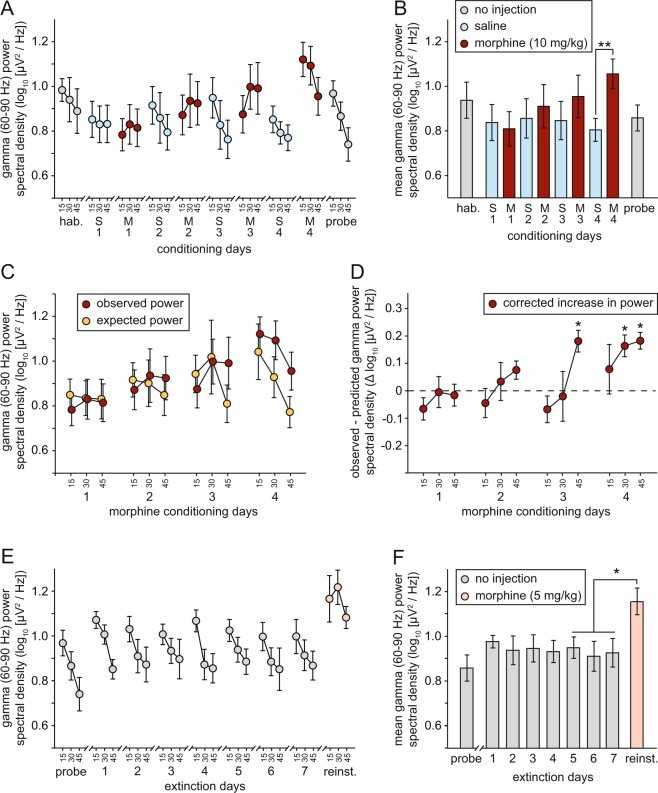
Morphine-induced sensitization of NAcS high-gamma activity (n = 7 throughout). (**A**) Gamma power spectral density during habituation (hab.), conditioning, and probe trials, divided into 15-min time bins (S1 = saline day 1; M1 = morphine day 1, etc.). (**B**) Mean gamma power spectral density during the whole 45 min of each of the trials plotted in A [*p < 0.01; paired-sample t-test with Benjamini-Hochberg correction for multiple comparisons]. (**C**) Observed power spectral density (divided into 15-min time bins) during morphine conditioning days, versus power predicted based on the positive relationship between locomotor activity and gamma power; see Fig. S1B. (**D**) Morphine-induced increase in gamma power corrected for locomotor activity. [*p < 0.05; one-sample t-tests with Benjamini-Hochberg correction for multiple comparisons]. (**E**) Gamma power spectral density during probe, extinction, and reinstatement (reinst.) trials, divided into 15-min time bins. (**F**) Mean gamma power spectral density during the whole 45 min of each of the trials plotted in (**E**) [*p < 0.05; paired-sample t-test].

The acquisition of CPP did not result in any significant long-term change in theta-frequency activity in the absence of drug administration, indicated by the lack of a difference between habituation and probe trials [gray circles in Fig. 6A and gray bars in Fig. 6B; F(1,6) = 0.02; p = 0.89]. The same was true for high-gamma activity [Fig. 7A,B; F(1,6) = 4.44; p = 0.08]. To assess any influence of the morphine- or saline-associated chamber on LFP activity during the probe trial, we used Any-maze tracking data to segregate LFP samples according to the chamber in which the rat was located at the time of sampling. This analysis focused only on the first half of the trial only (0–22.5 min), because 4/7 rats did not explore the saline-associated chamber at all during the second half of the trial. There was no difference between saline and morphine chambers in mean theta power [saline: 2.38 ± 0.09 log_10_(µV^2^/Hz); morphine: 2.41 ± 0.09 log_10_(µV^2^/Hz); t(6) = 1.11; p = 0.31] and high-gamma activity [saline: 0.96 ± 0.07 log_10_(µV^2^/Hz); morphine: 0.92 ± 0.06 log_10_(µV^2^/Hz); t(6) = 1.28; p = 0.25] over this period.

During conditioning, a widening difference emerged between morphine and saline days in theta activity (Fig. 6A,B; red and blue circles/bars). An ANOVA in which drug (morphine versus saline), testing day (saline days 1–4 & morphine days 1–4), and time (i.e. within-session 15-min time-window) were all entered as within-subjects factors revealed a significant overall difference in NAcS theta activity (7–12 Hz) between morphine and saline days [F(1,6) = 21.4; p = 0.004], and a significant drug x testing day interaction [F(3,18) = 9.73; p < 0.0005]. Across the four morphine days, there was a significant increase in theta activity [F(3,18) = 4.54; p = 0.015], whereas activity declined across saline days [F(3,18) = 4.24; p = 0.020]. Pairwise comparisons (paired-sample t-tests with Benjamini-Hochberg corrections for multiple comparisons) between all 4 morphine and saline days revealed significant differences in theta on day 3 [S3 versus M3 in Fig. 6B: t(6) = 3.63; p = 0.022] and day 4 [S4 versus M4 in Fig. 6A: t(6) = 3.68; p = 0.008]. In addition to the increase in theta power, there was a non-significant trend toward an increase in mean peak theta frequency between saline day 4 and morphine day 4 [saline: 8.06 ± 0.39 Hz; morphine: 9.25 ± 0.55 Hz; t(6) = 2.10; p = 0.08; cf. Fig. 5B].

A similar widening difference emerged between morphine and saline days in high-gamma activity (Fig. 7A,B; red and blue circles/bars). An ANOVA of mean NAcS high-gamma power (60–90 Hz) during conditioning days revealed a significant interaction of drug treatment and testing day [F(1.68,18) = 13.0; p < 0.002; Greenhouse-Geisser correction], with power increasing across successive morphine days [F(3,18) = 14.1; p = 0.0005] but remaining unchanged over successive saline days [F(3,18) = 0.48; p = 0.69]. Pairwise comparisons (paired-sample t-tests with Benjamini-Hochberg corrections for multiple comparisons) between all 4 morphine and saline days revealed a significant difference in high-gamma activity on day 4 [S4 versus M4 in Fig. 7B: t(6) = 3.69; p = 0.004].

Increases in low-gamma activity (30–45 Hz) were less pronounced (data not shown), with neither a main effect of morphine versus saline administration [F(1,6) = 1.40; p = 0.28], nor a significant drug x conditioning day interaction [F(1.36, 18) = 4.82; p = 0.051; Greenhouse-Geisser correction].

As theta and high-gamma power correlate with locomotor and exploratory activity, we calculated predicted power in both frequency bands on morphine days based on the relationship between distance traveled and LFP power on days without drug administration (Supplementary Information; Fig. S1), and the observed distance traveled after morphine administration (Fig. 3A). A difference between observed and expected power was evident in both theta and high-gamma bands (Figs 6C and 7C), and the corrected increase in power (i.e. observed - expected) increased both within conditioning sessions, and across conditioning days (Figs 6D and 7D; see Supplementary Information for statistical analysis). These data indicate that repeated administration of morphine leads to a sensitization of the theta and high-gamma-frequency response to drug administration, even after controlling for drug-induced increases in locomotion.

There were no significant changes across the probe trial and subsequent extinction days in theta power [Fig. 6E,F; F(7,42) = 1.66; p = 0.14] or high-gamma power [Fig. 7E,F; F(7,42) = 1.70; p = 0.14]. There was a significant increase between mean value during the final 3 extinction days and the reinstatement day in both frequency bands [theta: t(6) = 2.63; p = 0.04; high-gamma: t(6) = 3.17; p = 0.019]. However this increase was no greater than that predicted based on the locomotor increase after reinstatement (Fig. 3C; see Supplementary Information for details).

## Discussion

Consistent with previous reports, we find that electrical stimulation of the ventral CA1/subiculum evokes a multi-component evoked field potential (EFP) in the nucleus accumbens shell, with a prominent positive peak at a latency of 10–12 ms. Similar EFPs have been reported by others in response to stimulation of the ventral CA1/subiculum^36^, or stimulation of downstream afferents in the fimbria^22–25^. The initial positive peak was still evident at a test-pulse frequency of 50 Hz, and followed the stimulation pulses with a constant onset latency. This, together with the short latency of the response, supports the view that this component reflects monosynaptic transmission between the VH and NAcS. Further support for this view is provided by the observation that action potentials evoked by stimulation of hippocampal afferents are clustered around the rising phases and peaks of the positive-going components of the evoked field potential^22,24^. As others have reported, this pathway supports both short-term plasticity, such as paired-pulse facilitation (Fig. 1C^22^), and long-term synaptic changes such as tetanus-induced long-term potentiation (LTP; e.g.^23^). In contrast to the positive evoked responses reported in the NAc shell, negative evoked responses are observed in the core and/or at the dorsal and ventral margins of the NAc^22–25,36^; for examples of depth profiles, see^37^.

Consistent with the idea that synaptic plasticity underlies memory formation, learning-related increases in synaptic strength have been reported in many regions of the brain^38,39^. However, in the current study, the formation of a morphineconditioned place preference did not result in any changes in synaptic strength in the ventral CA1/subiculum projections to the shell of the nucleus accumbens. This was indicated by the equivalent EFP slope values recorded during habituation to the testing apparatus, a post-conditioning probe trial, and during and after extinction of the context–morphine reward association. There was also no difference on the probe trial between the size of EFPs recorded in the morphine versus saline-associated chambers of the apparatus, ruling out the possibility of a location-specific enhancement of the evoked response. At face value, this result does not support the idea that VH–NAcS synapses are likely sites for the storage of this form of memory. However, our findings stand in apparent contrast to those of a recent study in which high-frequency optogenetic stimulation of vHPC-NAc synapses was sufficient to induce a place preference in mice that had not undergone CPP training^28^. However, in contrast to high-frequency stimulation induced potentiation, is possible that natural learning-related changes occur sparsely or selectively in a small number of inputs, such as those afferents originating in the place cells that encode the rewarded location (see^34^), or that simultaneous increases and decreases in synaptic strength mask any overall change. For example, it has been found that repeated morphine administration causes an increase in synaptic strength *ex vivo* in glutamatergic inputs to dopamine D1-receptor-expressing MSNs, but a decrease in synaptic strength at inputs to D2-expressing MSNs^35^. However, it has been reported that ventral hippocampal afferents make weaker synaptic connections with D2- compared to D1-receptor expressing MSNs^40^. Although there is evidence that interactions between the hippocampus and nucleus accumbens shell are involved in the association of environmental context with reward (e.g.^27,41^), we lack a detailed understanding of the way in which this information is represented at the level of individual glutamatergic afferents, their termination patterns, and their neurons of origin. In addition, there is evidence in area CA1 of the hippocampus that morphine CPP can cause an increase in basal synaptic transmission^42^ and dendritic remodeling^43,44^. In other words, some aspects of the relevant learning-related plasticity may occur upstream in the hippocampus, before being relayed to the accumbens.

It is also possible that the relevant plasticity occurs in other glutamatergic pathways. For example, the medial prefrontal cortex—another structure implicated in the acquisition of conditioned place preference^45^—sends extensive glutamatergic projections to the NAc; these afferents are plastic, and exhibit competitive and cooperative interactions with hippocampal inputs^36,46^. Afferents from both structures may converge at the level of individual MSNs^47^. Recent evidence suggests that plasticity in projections from the infralimbic region of the prefrontal cortex is involved in morphine-associated place preference^35^. It would therefore be interesting to examine whether learning-related changes are evident in evoked field potentials recorded in this pathway during the acquisition and extinction of CPP.

Another possibility is that cocaine and opioid-induced CPP engage distinct neural mechanisms. There is consistent evidence that repeated cocaine administration enhances basal synaptic strength in the VH–NAc projection in *ex vivo* hippocampal slices, and a recent study provides evidence that cocaine-induced CPP leads to a place-specific strengthening of the coupling between the activity of hippocampal place cells representing the reward location, and D2-containing MSNs of the NAc^34^ (although Calipari and colleagues^48^, have reported increases in D1 MSN activity in association with the reward location). In contrast, morphine CPP has been reported to cause a decrease in the activity of MSNs in response to the rewarded context^49^, suggesting that associations between opioid reward and context may be represented differently. However, in the latter case, the source of the afferents driving the decrease in firing is not known.

Despite the absence of learning-related changes in the present experiment, morphine administration caused an acute increase in the size of the accumbens EFP. An analysis of the time-course of the effect in a separate group of rats revealed a maximal increase at around 45 min after injection, and a complete return to baseline within 24 h. This effect was evident the very first time morphine was administered to each rat, and remained constant with successive injections; the absence of sensitization suggests an acute effect of the drug, rather than an association between context and drug reward. Although increased locomotor activity can cause a rise in brain temperature that increases the slope of EFPs^50^, this phenomenon cannot account for the morphine-induced changes in NAcS EFPs observed here. First, the increase was evident even after the first injection, when locomotor activity was slightly below baseline levels. And second, the EFP increase was maximal at the end of a session, even after activity levels had fallen to baseline values or below. In hippocampal area CA1 *in vivo*, morphine also causes an enhancement of the EFP (e.g.^51,52^); this effect is usually attributed to a reduction in GABA-ergic transmission and the disinhibition of pyramidal cells^53^, analogous to the µ-opioid-dependent disinhibition of VTA neurons that underlies the rewarding effects of morphine (e.g.^54^). However, µ-opioid receptor activation may also have direct effects on fast synaptic transmission by increasing the synaptic expression of GluA1-containing AMPA receptors^35,55^.

In contrast to the constant magnitude of the EFP increase induced by successive injections of morphine, and the absence of learning-related changes, the theta and gamma frequency LFP response to morphine became markedly sensitized with repeated administration, as did the locomotor response to the drug. Locomotor sensitization is often observed following repeated opioid administration, and a component of the response may be context-dependent and selective for the reward-associated environment. The phenomenon depends, in part, on plasticity in the response of the mesolimbic dopamine system (see^56^), although changes in AMPA-mediated transmission may also play a role^57^. At the dose used in the present study (10 mg/kg), rats typically exhibit a small depression of activity after an initial injection of morphine, with increases in motor activity occurring with subsequent drug exposure. Consistent with this, we found a progressive increase in locomotor activity with successive injections of morphine, relative to days on which saline was administered. This increase was most pronounced during the first 15 min of the trial, and activity levels returned to saline levels or below by the end of each session.

In parallel to the locomotor sensitization, we also observed a progressive increase in the power of accumbens theta and high-gamma-frequency activity with repeated drug administration. Similar increases were observed in the ventral hippocampus, consistent with reports of coherent activity in the two structures, particularly at theta frequencies^26,58,59^. A potential confound is that both gamma and theta activity increase in tandem with locomotor activity under baseline conditions in the absence of drug. However, an examination of the observed versus predicted increases in theta and gamma power revealed that the morphine-induced increase was in both cases larger than that predicted by the locomotor increase alone. The time course of the LFP changes was also different—increases in theta and gamma power were typically maintained for the duration of a session, long after locomotor activity levels fell to baseline levels or below. Pharmacokinetic data indicate that the brain concentration of morphine peaks about 45 min after subcutaneous injection in the rat—i.e. the end of the conditioning session in the present study. It is therefore possible that the observed LFP changes parallel the time-course of drug action, unlike the locomotor response that declines more rapidly over time.

The sensitized increase in NAcS gamma-frequency power was most pronounced in the high-gamma range (60–90 Hz), whereas changes in low-gamma activity were less prominent. Our results differ slightly from those of a previous study of the acute effects of morphine exposure on NAc LFP responses, in which increases in both low and high-frequency gamma were observed after a single dose^60^. However, this study was conducted in mice, and the apparent discrepancy may reflect the acute locomotor stimulant effect of morphine in this species, in contrast to rats in which hypolocomotion is typically observed when morphine is first administered. Previous studies involving food reward have indicated that high gamma activity is associated with reward expectancy, whereas low gamma is associated with reward delivery^61^. For example, we have observed that mice trained to make a nose-poke response for food reward exhibit an increase in approximately 70-Hz activity in the NAc immediately after the response (DY Sakae, B Pais, & SJ Martin, unpublished observations). The locomotor sensitization that follows repeated opioid administration has often been linked to the increased drug-seeking behavior, drug craving, and association of environmental cues with drug reward, that occur after multiple drug exposures, changes that may be related to the development of addiction (cf.^62,63^). The morphine-induced sensitization of high-gamma activity—a frequency associated with reward expectancy in studies of natural reward—may provide an additional index of this incentive-sensitization process, and one that is not directly tied to the animal’s ongoing behavior.

## Materials and Methods

### Animals

All experiments were conducted under a UK Home Office Project Licence (70/8517), in accordance with the Animals (Scientific Procedures) Act, 1986, and the European Communities Council Directive of 24 November 1986 (86/609/EEC). The programme of work and procedures used were approved by the University of Dundee’s *Welfare and Ethical Use of Animals Committee*. This committee reports to the University Court and discharges the responsibility of an Animal Welfare and Ethical Review Body, as mandated under the Animals (Scientific Procedures) Act.

Male Lister-hooded rats, 250–400 g at the start of the experiment, were purchased from Charles River, UK. They were housed in pairs with ad libitum access to standard rat chow and water, and a 12-h light/dark cycle. Home-cage dimensions were 32 × 50 cm; woodchip bedding and paper nesting material were provided, as well as wood sticks for chewing, and enrichment items such as cardboard or plastic tubes. Rats were acclimatized to the animal unit for 2 weeks before the start of the experiment, during which they were handled regularly. All experiments were conducted during the light phase.

### Drugs

Morphine sulphate (10 mg/kg; Sigma-Aldrich, Gillingham, UK) was dissolved in sterile physiological saline (0.9% w/v); it was injected subcutaneously at a volume of 1 ml/kg.

### Behavioral apparatus

The CPP apparatus comprised two contiguous open-topped acrylic chambers, 26 × 30 cm at the base, and 40-cm tall, connected by a 10-cm-wide doorway that could be closed when required using a ‘guillotine-style’ door insert. The two chambers contained distinctive tactile and visual features. The left chamber of the apparatus comprised black-and-white striped walls, and a removable ridged acrylic floor. The right chamber comprised uniform 50% gray walls and smooth flooring.

The entire apparatus was enclosed in a ventilated recording cabinet, with an extraction fan for air circulation and background noise, and diffuse lighting providing a light level of approximately 90 lux within the CPP apparatus. To minimize electrical interference, the interior walls of the recording cabinet were coated with grounded aluminium foil secured behind plastic-coated wooden panels. A 12-channel electrical commutator (Adafruit Industries, New York City, USA) was mounted on the ceiling of the cabinet, and a 12-channel Plastics One recording cable (Bilaney, Sevenoaks, UK) was connected to the commutator for tethered stimulation and recording from freely moving rats. This cable was attached to a ceiling-mounted spring that prevented unwanted cable movement and slack during movement of the animal. The commutator was connected to the stimulation and recording system (see Supplementary Information) via a BNC break-out board and BNC cables.

The rats’ behavior was monitored via a CCTV board camera (Henry’s Electronics, London, UK) mounted alongside the commutator, and connected to an Adlink RTV-24 video-capture card (Amplicon, Brighton, UK) in a PC running Any-maze software (Stoelting Europe, Dublin, Ireland) for the capture and analysis of locomotor activity.

### Surgery and electrode implantation

Recording electrodes comprised two twisted strands of PTFE-insulated platinum/iridium wire (uncoated diameter = 0.075 mm), with a vertical separation of ~0.2 mm between the exposed tips of the two wires. Stimulating electrodes comprised three twisted stands of the same wire, again with a vertical separation of ~0.2 mm between electrode tips. Before implantation, electrodes were soldered to contact pins for later insertion into an electrode pedestal. The use of multi-wire electrodes provided a choice of recording and stimulation channels in the event of post-surgical movement of the electrodes.

Preliminary experiments were conducted under terminal urethane anesthesia (ethyl carbamate 1.5 g/kg; 0.3 mg/ml IP) (n = 2; see Fig. 1). Stimulating and recording electrodes were mounted on stereotaxic manipulators for acute placement and connected directly to the stimulus isolator boxes and amplifier. LFP data were not collected. Other details were the same as those outlined below.

For recovery surgery, anesthesia was induced and maintained using isoflurane. Rats (n = 3 for the morphine time-course experiment; n = 8 for the CPP experiment) were positioned in a stereotaxic frame (Kopf, Tujunga, CA, USA) with head horizontal. Under aseptic conditions, an incision was made in the skin and periosteum of the skull, small burr-holes were made above the stimulation and recording sites, and the dura was pierced with a sterile needle. Stimulating and recording electrodes were then implanted bilaterally under stereotaxic guidance, targeting the ventral CA1/subiculum region of the hippocampal formation (coordinates relative to bregma: AP = −6.0 mm; Lat. = ± 5.2 mm; DV from dura ~−7.0–7.5 mm) and the medial shell of the nucleus accumbens (AP = 1.7 mm; Lat. = ± 0.8 mm; DV from dura ~−6.5 mm). The DV position of each electrode was finalized by maximizing the amplitude of a characteristic multi-component EFP response (see below) elicited in the NAcS by ventral CA1/subiculum stimulation. After implantation, the electrode contact pins were inserted into a 12-channel electrode pedestal (Plastics One, Bilaney, Sevenoaks, UK) for later connection to a recording cable. The electrodes and pedestal were then secured to the skull using stainless steel micro-screws and dental cement. Two of these screws (secured to the occipital bone) served as cortical references (one primary and one spare) for differential recording of LFP and EFP responses; these screws were also connected to contact pins inserted into the electrode pedestal. After implantation, the electrode pedestal was covered with a dust-cap to protect the electrode contacts. Rats were monitored closely during postoperative recovery; carprofen (Rimadyl small animal solution, 4 mg/kg; SC), was administered for analgesia upon anesthetic induction as well as postoperatively.

### LFP recording

For each channel, the continuous broad-band local field potential (LFP) was amplified and filtered (high pass = 1 Hz; low pass = 5 kHz) using a 16-channel differential AC amplifier (Model 3500, A-M Systems, Sequim, WA, USA), and sampled at 20 kHz using a data acquisition card (PCIe-6321; National Instruments, Austin, TX, USA) mounted in a PC running custom-written LabView software for LFP capture and analysis developed by Patrick Spooner (University of Edinburgh). Automatic selection of a 2-s sample of the raw LFP trace was triggered to occur 60 s after the onset of an EFP (to prevent the contamination of LFP with evoked activity). Using the same software, each sample was temporally filtered using a Hanning window to prevent onset/offset artifacts, bandpass filtered between 0.5 and 200 Hz, notch filtered at 50 Hz to remove mains interference, and spectrally analyzed using the fast Fourier transform (FFT) algorithm. This resulted in a series of spectral plots for each recording session in which power spectral density was expressed as a function of frequency from 0–100 Hz, divided into 0.5-Hz bins. These data were then converted to log_10_ values, and mean power was calculated over three 15-min time windows for each 45-min recording session for specific frequency ranges, including theta (7–12 Hz) and high-gamma (60–90 Hz).

### Evoked field potential (EFP) recording

The output of the amplifier was also connected to a separate data acquisition card and PC running software for the control of electrical stimulation and the time-locked recording of evoked EFPs sampled from the continuous LFP trace (Evoked Potential Sampler, Patrick Spooner, University of Edinburgh). This program calculates a range of field potential measures, such as amplitude and slope (measured by linear regression between two fixed time-points); in this study, we focus on the slope of the rising phase of the first positive component of the EFP, measured over a 1.5-ms time-window, typically starting between 6.0–8.0 ms after stimulation. Stimulation was delivered via a NeuroLog system and stimulus-isolator units (DS4; Digitimer, Welwyn Garden City, UK), and consisted, during the main experiment, of biphasic constant-current pulses (1.0 mA; 0.2 µs per phase) delivered every 2 min. An Arduino Uno controller (Arduino, Turin, Italy) connected to relay switches and running timing software was used to switch the hippocampal electrode channels between recording and stimulation configurations during the delivery of test pulses.

During initial screening sessions, rats were habituated to connection of the recording cable and the optimal combination of stimulation and recording channels was selected. During these sessions, rats were placed in a separate environment to the CPP apparatus—the base of a standard holding cage with woodchip bedding. Although stimulating and recording electrodes were implanted bilaterally, the hemisphere that yielded the largest EFPs with an expected response latency and shape was selected for stimulation and recording of evoked EFPs. Within this hemisphere, the two (out of a total of three) hippocampal stimulation channels that yielded the best evoked EFPs were selected for bipolar stimulation in the main experiment; the ‘best’ recording channel (out of two) based on lack of interference and EFP characteristics was also chosen. One of the selected stimulation channels and the selected recording channel were connected to Humbug Noise Eliminators (Digitimer, Welwyn Garden City, UK) for the reduction of electrical interference. Subsequent analysis focused on these two channels. Of the 8 animals implanted for the CPP experiment, 1 failed to show NAcS EFPs during screening and was removed from the study, leaving n = 7 throughout the main experiment.

### Behavioral testing

After a 2-week post-surgery recovery period, and the initial screening session detailed above, rats underwent CPP training (Fig. 2A) while EFP and LFP data were collected (see Supplementary Information for further details). This began with a single 45-min habituation session during which the rats could explore both chambers of the apparatus with the door open. This was followed by 8 days of conditioning in which each rat was confined to one chamber of the apparatus for 45 min immediately following saline injection, and the opposite chamber following morphine injection the next day. This was repeated for 8 days, with saline and morphine injections administered on alternating days. The use of a single daily injection and recording session was chosen to avoid potential diurnal variations in EFP, LFP, and baseline locomotor activity. The choice of drug associated with each chamber was counterbalanced, and preferences for drug and saline sides were matched across animals based on exploration during habituation. After the conditioning phase, rats received a 45-min probe trial in which they could explore both chambers; this was identical to the habituation trial. After a 1-week period during which rats received no drug administration or exposure to the apparatus, animals were tested during extinction for another 7 days; these sessions comprised a 45-min exploration of the whole apparatus (identical to the habituation and probe trials), and no injections were given. The reinstatement test was also identical to the extinction trials except that a 5 mg/kg dose of morphine was injected immediately before the trial.

### Histology

At the end of each experiment, marking lesions were made under terminal pentobarbitone anesthesia by the delivery of biphasic 1-mA constant-current pulses (1 s per phase) delivered to the chosen recording and stimulation channels explained above. Post mortem, brains were removed and stored in 10% formalin. 30-µm coronal sections were then cut using a cryostat. Slide-mounted sections were stained with cresyl violet and examined under a light microscope. Stimulation and recording sites were identified and recorded on the appropriate coronal section of the Paxinos and Watson (2004) atlas^64^, based on electrode tracks and marking lesions. All recording electrodes were located in the NAcS (Fig. 1E), and all stimulating electrodes were located in ventral CA1 or subiculum (Fig. 1F).

### Statistics

Statistical analysis was carried out using SPSS. In all cases in which multiple pairwise comparisons or one-sample t-tests were conducted on the same set of data, the Benjamini-Hochberg procedure was used to calculate an adjusted p value, with the false discovery rate set to 0.05. Two-tailed tests were applied in all cases.

## Supplementary information

Sakae & Martin 2019 - Supplementary Information

## Data Availability

The datasets generated during and/or analysed during the current study are available from the corresponding author on reasonable request.
