# Supplementary material for: Formation of a morphine-conditioned place preference does not change the size of evoked potentials in the ventral hippocampus–nucleus accumbens projection
Source: Sci Rep. 2019 Mar 26;9:5206. doi: 10.1038/s41598-019-41568-5 (PMC6435809; doi:10.1038/s41598-019-41568-5)
Supplement: Supplementary file 1 — Sakae & Martin 2019 - Supplementary Information [file 41598_2019_41568_MOESM1_ESM.pdf]

Formation of a morphine-conditioned place preference does not change the size of evoked potentials in the ventral hippocampus–nucleus accumbens projection.

Supplementary Information

Supplementary Results

Morphine-induced theta-frequency LFP sensitization in the NAcS (Fig. 6): correction for locomotor activity

Theta power correlated significantly with locomotor activity. Mean theta power during each 15-min time window during habituation, probe, and saline days was plotted against distance traveled over the same period for each rat (Fig. S1A). Individual correlation coefficients were converted to z-scores using Fisher's r-to-z transformation, before averaging and conversion back to an overall r value [$r = 0.56$; $p = 0.016$]. We then calculated a predicted theta power on morphine days based on the relationship between distance travelled and theta power on days without drug administration. Based on the slope and y-intercept values obtained, and the observed locomotor activity recorded during each 15-min window on morphine days (Fig. 3A), we calculated predicted theta power values, and plotted them against observed values (Fig. 6C). There was a significant interaction between observed / predicted values and within-session time-point [$F(2,12) = 13.50$; $p = 0.001$]. Considering the last 15-min of each session only, the point at which the difference between predicted and observed values was largest, observed values were significantly higher than predicted values [$F(1,6) = 6.28$; $p = 0.046$], with a significant interaction between observed / predicted values and testing day [$F(3,18) = 3.59$; $p = 0.034$], reflecting the widening difference between predicted and observed values over days.

We then calculated corrected theta values, with the influence of locomotor activity removed, by subtracting predicted values for observed values at each time point for each rat (Fig. 6D). Corrected theta power increased significantly within testing sessions [$F(2,12) = 13.5$; $p = 0.001$], although the overall increase across testing days did not reach significance [$F(3,18) = 2.28$; $p = 0.11$]. Post-hoc

comparisons of corrected theta power with chance were conducted across all days and time points (one-sample t-tests with Benjamini-Hochberg corrections for multiple comparisons). Corrected theta activity was significantly elevated during the final 15 min of day 4 [$t(6) = 4.54$; adjusted $p = 0.047$]. The observed increase in theta activity at this point (Fig. 6D) corresponds to an approximate doubling of theta power relative to the predicted value.

Although there was a significant increase in NAcS theta between mean value during the final 3 extinction days and the reinstatement day in both frequency bands (see main Results section), this increase was no different to that predicted based on the locomotor increase after reinstatement [Fig 3C; mean predicted theta during reinstatement trial = 2.72 ± 0.27 $\log_{10}(\mu V^2/Hz)$; mean observed theta during reinstatement trial = 2.72 ± 0.13 $\log_{10}(\mu V^2/Hz)$; $t(6) = 0.006$; $p = 0.995$].

Morphine-induced gamma-frequency LFP sensitization in the NAcS (Fig. 7): correction for locomotor activity

High-gamma power, like theta, correlated significantly with locomotor activity. Mean theta power during each 15-min time window during habituation, probe, and saline days was plotted against distance traveled over the same period for each rat (Fig. S1B). Individual correlation coefficients were converted to z-scores using Fisher's r-to-z transformation, before averaging and conversion back to an overall r value [$r = 0.62$; $p = 0.006$]. We calculated a predicted high-gamma power on morphine days based on the relationship between distance travelled and high-gamma power on days without drug administration, as described for theta above. There was a significant interaction between observed / predicted values and morphine conditioning day [Fig. 7C; $F(3,18) = 5.56$; $p = 0.007$], and a significant interaction between observed / predicted values and within-session time-point [$F(2,12) = 5.46$; $p = 0.021$]. Considering the last 15-min of each session only, the point at which the difference between predicted and observed values was largest, observed values were very significantly higher than predicted values [$F(1,6) = 119.8$; $p = 0.004$], with a significant interaction between observed / predicted values and testing day [$F(3,18) = 9.24$; $p = 0.001$], reflecting the widening difference between predicted and observed values over days. Overall, observed values were significantly higher than predicted values on day 4 [$F(1,6) = 14.9$; $p = 0.008$].

We then calculated a corrected high-gamma power, as described for theta above (Fig. 7D). There was a significant increase in corrected power across days [$F(3,18) = 5.56$; $p = 0.007$], and also within

testing sessions [F(2,12) = 5.46; p = 0.021]. Post-hoc comparisons of corrected theta power with chance were conducted across all days and time points (one-sample t-tests with Benjamini-Hochberg corrections for multiple comparisons). Time points at which corrected high-gamma activity was significantly elevated are indicated on Fig. 7D. These data indicate that repeated administration of morphine during CPP training leads to a sensitization of the high-gamma-frequency response to drug administration in the NAcS, even after controlling for drug-induced increases in locomotion.

Although there was a significant increase in NAcS high-gamma between mean value during the final 3 extinction days and the reinstatement day in both frequency bands (see main Results section), this increase was no different to that predicted based on the locomotor increase after reinstatement [Fig 3C; mean predicted high-gamma during reinstatement trial = 1.22 ± 0.21 $\log_{10}(\mu V^2/Hz)$; mean observed high-gamma during reinstatement trial = 1.15 ± 0.06 $\log_{10}(\mu V^2/Hz)$; t(6) = 0.40; p = 0.70].

Morphine-induced theta-frequency LFP changes in the ventral hippocampus

The acquisition of CPP did not result in any significant long-term change in VH theta-frequency activity in the absence of drug administration, indicated by the lack of a difference between habituation and probe trials [gray circles in Fig. S3A and gray bars in Fig. S3B; F(1,6) = 0.32; p = 0.59].

An ANOVA of mean VH theta power (7-12Hz) during conditioning days (Fig. S3A & B) revealed a significant interaction of drug treatment and testing day [F(1.32,18) = 5.21; p = 0.046; Greenhouse-Geisser correction], with power increasing across successive morphine days [F(3,18) = 4.54; p = 0.015] but remaining unchanged over successive saline days [F(1.58,18) = 3.0; p = 0.11; Greenhouse-Geisser correction]. Pairwise comparisons (paired-sample t-tests with Benjamini-Hochberg corrections for multiple comparisons) between all 4 morphine and saline days revealed significant differences in theta on day 3 [S3 versus M3 in Fig. S3B: t(6) = 3.62; p = 0.022] and day 4 [S4 versus M4 in Fig. S3B: t(6) = 5.94; p = 0.004].

VH theta power correlated strongly with locomotor activity. The relationship between distance travelled and theta power on days without drug administration is plotted in Fig. S2A. Individual correlation coefficients were converted to z-scores using Fisher's r-to-z transformation, before averaging and conversion back to an overall r value [r = 0.52; p = 0.027]. Based on these correlations, we calculated a predicted theta power on morphine as described for the NAcS above. There was a

significant overall difference between observed and predicted values [Fig S3C; $F(1,6) = 17.0$; $p = 0.006$], and an interaction between observed / predicted values and within-session time-point [$F(1.16,12) = 13.5$; $p = 0.007$; Greenhouse-Geisser correction]. Considering the middle 15-min time-period of each session only, observed values were very significantly higher than predicted values [$F(1,6) = 11.7$; $p = 0.014$]; the same was true of the final 15-min of each session [$F(1,6) = 28.4$; $p = 0.002$].

We then calculated a corrected theta power, as described for the NAcS above (Fig. S3D). There was a significant increase in corrected power within testing sessions [$F(2,12) = 13.5$; $p = 0.001$], but the increase over days did not reach significance [$F(3,18) = 2.16$; $p = 0.13$]. Post-hoc comparisons of corrected theta power with chance were conducted across all days and time points (one-sample t-tests with Benjamini-Hochberg corrections for multiple comparisons).Time points at which corrected high-gamma activity was significantly elevated are indicated on Fig. S3D.

There were no significant changes across the probe trial and subsequent extinction days in theta power [Fig. S3E & F; $F(7,42) = 1.82$; $p = 0.11$]. However, there was a significant increase between mean value during the final 3 extinction days and the reinstatement day [$t(6) = 3.13$; $p = 0.02$], although this increase was not significantly greater than that predicted based on the locomotor increase after reinstatement [Fig 3C; mean predicted theta during reinstatement trial = 2.48 ± 0.12 $\log_{10}(\mu V^2/Hz)$; mean observed theta during reinstatement trial = 2.71 ± 0.12 $\log_{10}(\mu V^2/Hz)$; $t(6) = 1.90$; $p = 0.11$].

Overall, these results indicate that administration of morphine during CPP training causes an increase in VH theta, even after controlling for drug-induced increases in locomotion. However, the sensitization of this effect with repeated administration was less pronounced than that observed for VH high-gamma activity (see below), as indicated by the lack of a significant drug x day interaction.

Morphine-induced high-gamma-frequency LFP sensitization in the ventral hippocampus

The acquisition of CPP did not result in any significant long-term change in VH high-gamma-frequency activity in the absence of drug administration, indicated by the lack of a difference between habituation and probe trials [gray circles in Fig. S4A and gray bars in Fig. S4B; $F(1,6) = 0.998$; $p = 0.36$].

An ANOVA of mean high-gamma power (60-90 Hz) during conditioning days (S4A & B) revealed a significant overall morphine-induced increase [$F(1,6) = 8.47$; $p = 0.027$], and an interaction of drug treatment and testing day [$F(3,18) = 9.97$; $p < 0.0005$], with power increasing across successive morphine days [$F(3,18) = 26.0$; $p < 0.0005$] but remaining unchanged over successive saline days [$F(3,18) = 0.32$; $p = 0.81$]. Pairwise comparisons (paired-sample t-tests with Benjamini-Hochberg corrections for multiple comparisons) between all 4 morphine and saline days revealed significant differences in high-gamma power on day 4 [S4 versus M4 in Fig. S4B: $t(6) = 3.68$; $p = 0.04$].

VH high-gamma power, like theta, correlated strongly with locomotor activity. The relationship between distance travelled and high-gamma power on days without drug administration is plotted in Fig. S2B. Individual correlation coefficients were converted to z-scores using Fisher's r-to-z transformation, before averaging and conversion back to an overall r value [$r = 0.74$; $p = 0.0004$]. Based on these correlations, we calculated a predicted high-gamma power on morphine days as described for the NAcS above. There was a significant interaction between observed / predicted values and morphine conditioning day [Fig. S4C; $F(3,18) = 11.3$; $p < 0.0005$]. Overall, observed values were significantly higher than predicted values on day 4 [$F(1,6) = 19.4$; $p = 0.005$].

We then calculated a corrected high-gamma power, as described for the NAcS above (Fig. S4D). There was a significant increase in corrected power across days [$F(3,18) = 11.3$; $p < 0.0005$]. Post-hoc comparisons of corrected theta power with chance were conducted across all days and time points (one-sample t-tests with Benjamini-Hochberg corrections for multiple comparisons).Time points at which corrected high-gamma activity was significantly elevated are indicated on Fig. S4D.

There were no significant changes across the probe trial and subsequent extinction days in theta power [Fig. S4E & F; $F(7,42) = 1.78$; $p = 0.12$]. However, there was a significant increase between mean value during the final 3 extinction days and the reinstatement day [$t(6) = 4.04$; $p = 0.007$], although this increase was not significantly greater than that predicted based on the locomotor increase after reinstatement [Fig 3C; mean predicted high-gamma during reinstatement trial = $0.90 \pm 0.05 \log_{10} (\mu V^2/Hz)$; mean observed high-gamma during reinstatement trial = $0.96 \pm 0.07 \log_{10} (\mu V^2/Hz)$; $t(6) = 1.52$; $p = 0.18$].

These results show that repeated administration of morphine during CPP training causes a pronounced sensitization of the high-gamma-frequency response to drug administration in the VH, even after controlling for drug-induced increases in locomotion.

Supplementary Figures

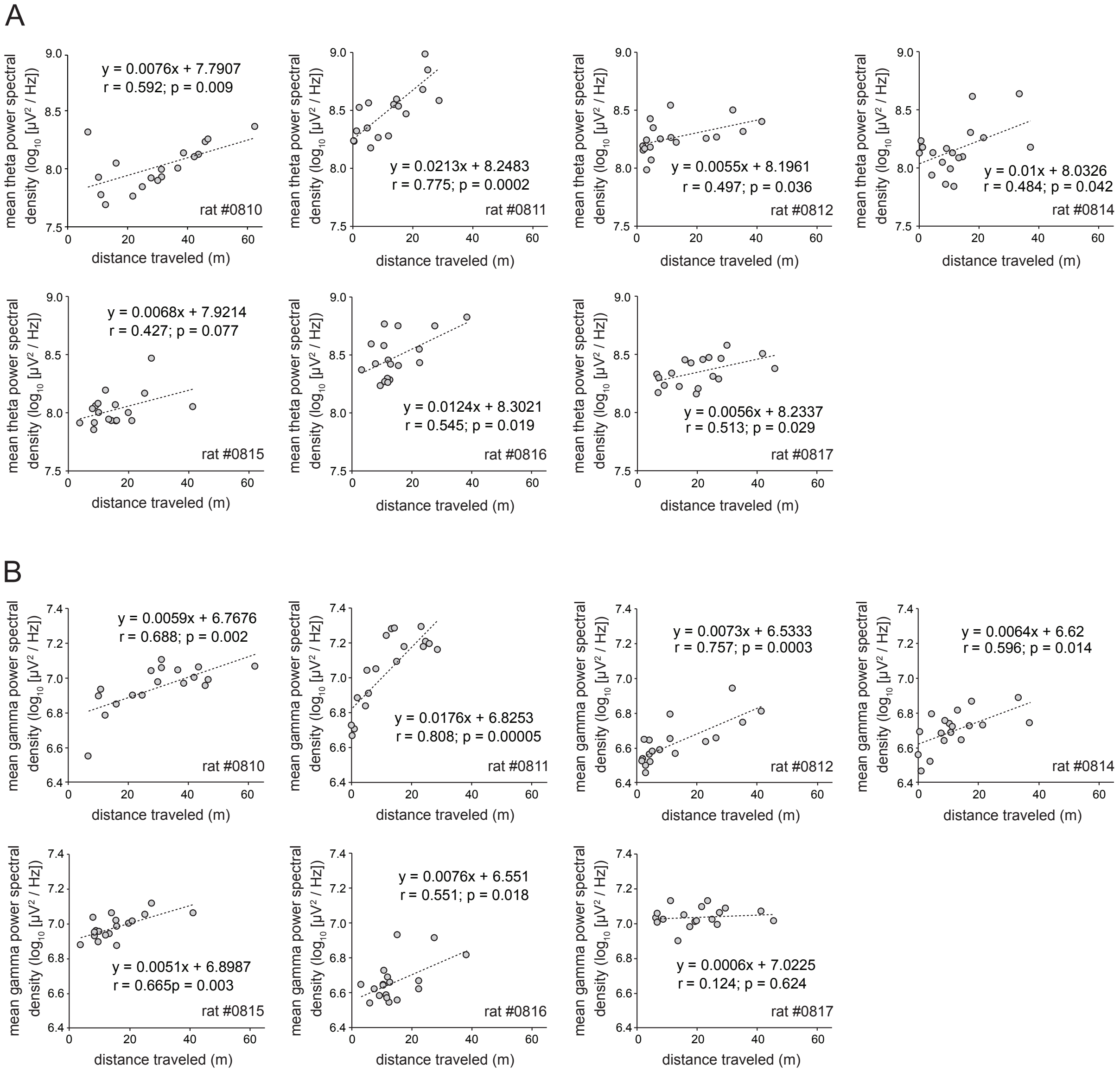

Figure S1. Correlations between NAcS LFP power and locomotor activity. Theta (A) and high-gamma (B) power spectral density as a function of distance traveled in the absence of morphine. Each plot represents data from a single rat, and individual data points represent the means of each 15-min recording period during habituation, saline conditioning, and probe trials.

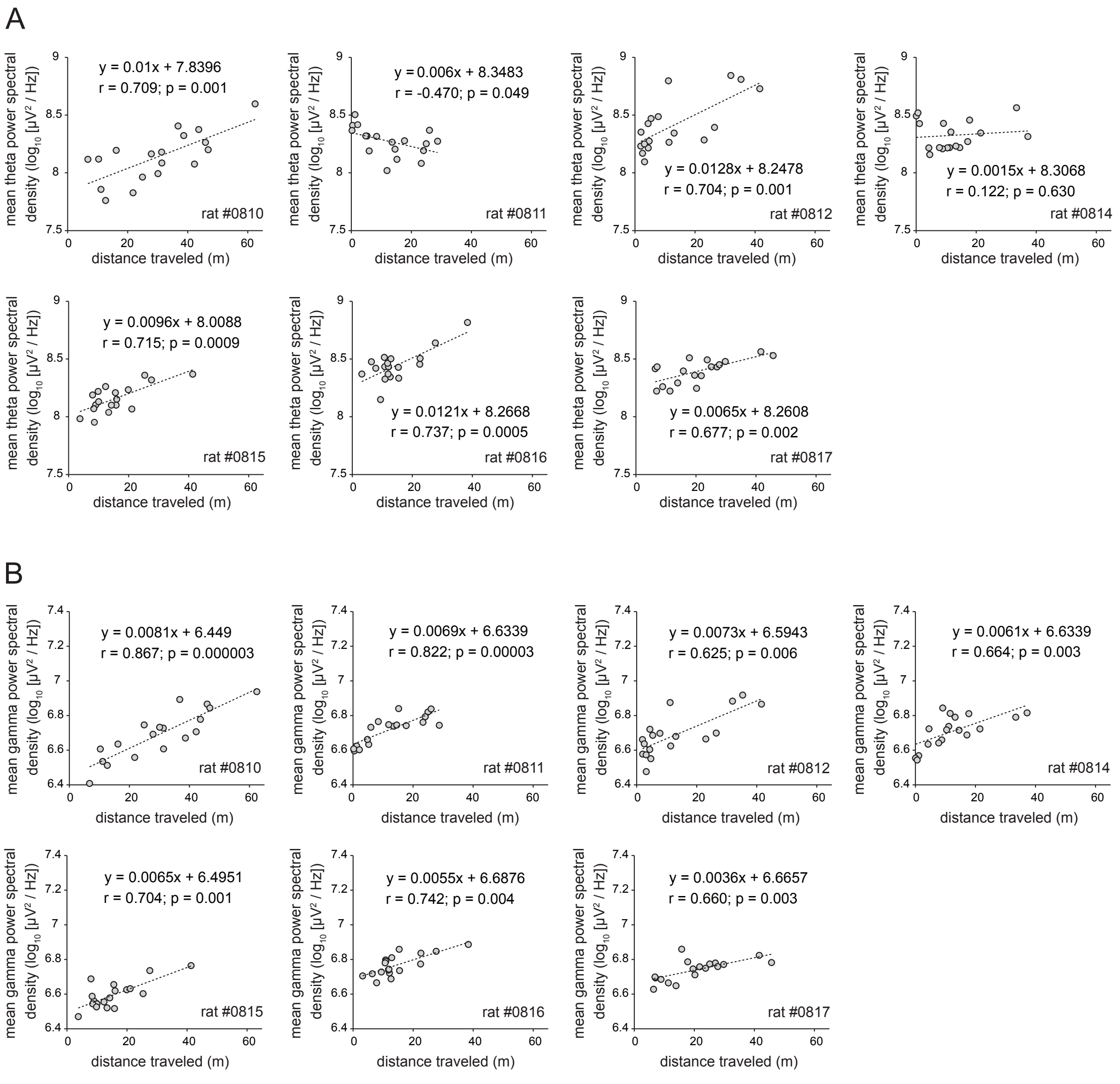

Figure S2. Correlations between ventral hippocampal LFP power and locomotor activity. Theta (A) and high-gamma (B) power spectral density as a function of distance traveled in the absence of morphine. Each plot represents data from a single rat, and individual data points represent the means of each 15-min recording period during habituation, saline conditioning, and probe trials.

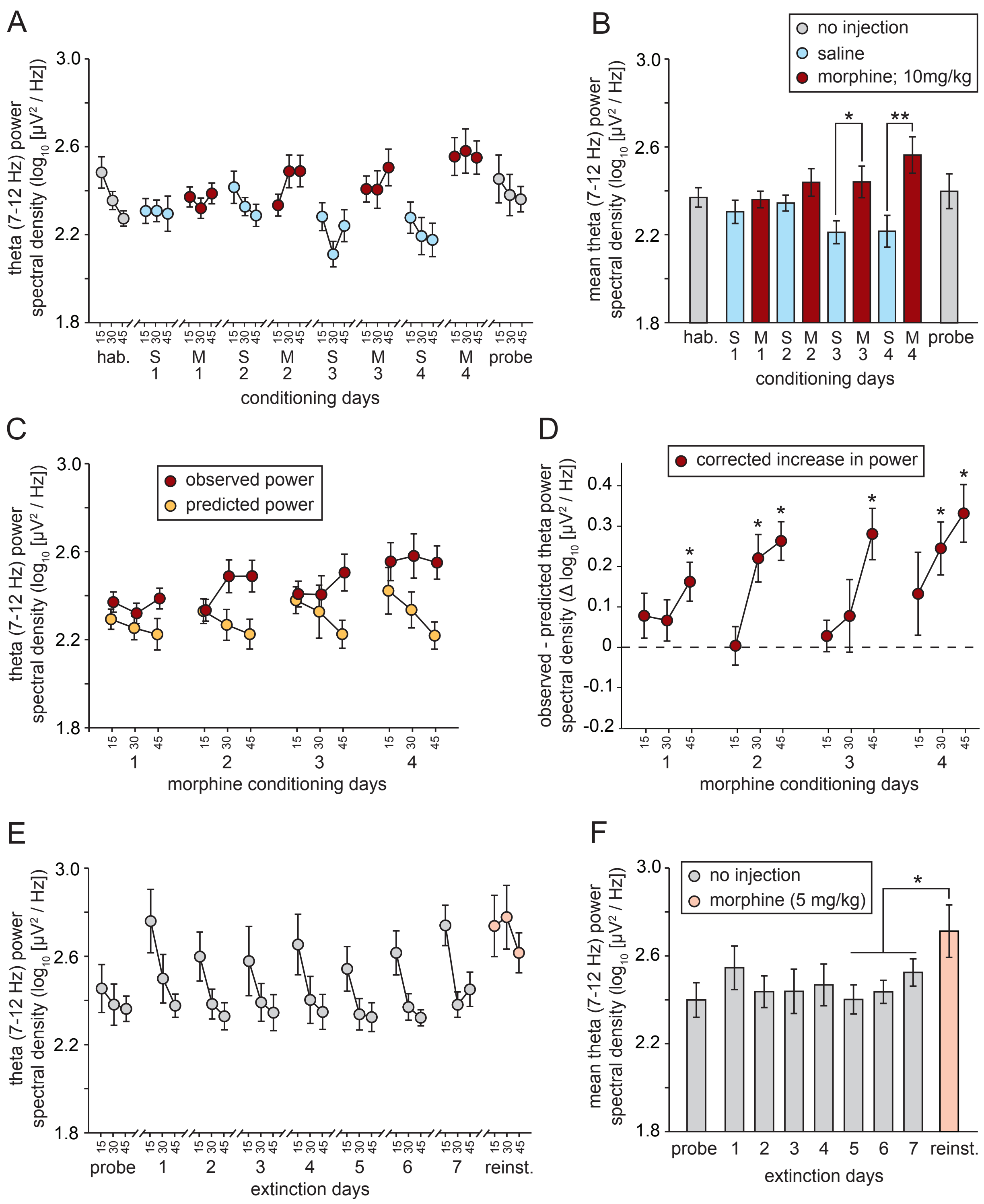

Figure S3. Morphine-induced sensitization of ventral hippocampal theta activity. (A) Theta power spectral density during habituation (hab.), conditioning, and probe trials, divided into 15-min time bins (S1 = saline day 1; M1 = morphine day 1, etc.). (B) Mean theta power spectral density during the whole 45 min of each of the trials plotted in A [*p < 0.05; **p < 0.01; paired-sample t-tests with Benjamini-Hochberg correction for multiple comparisons]. (C) Observed power spectral density (divided into 15-min time bins) during morphine conditioning days, versus power predicted based on the positive relationship between locomotor activity and theta power; see Fig. S2A. (D) Morphine-induced increase in theta power corrected for locomotor activity [*p < 0.05; one-sample t-tests with Benjamini-Hochberg correction for multiple comparisons]. (E) Theta power spectral density during probe, extinction, and reinstatement (reinst.) trials, divided into 15-min time bins. (F) Mean theta power spectral density during the whole 45 min of each of the trials plotted in E [*p < 0.05; paired-sample t-test].

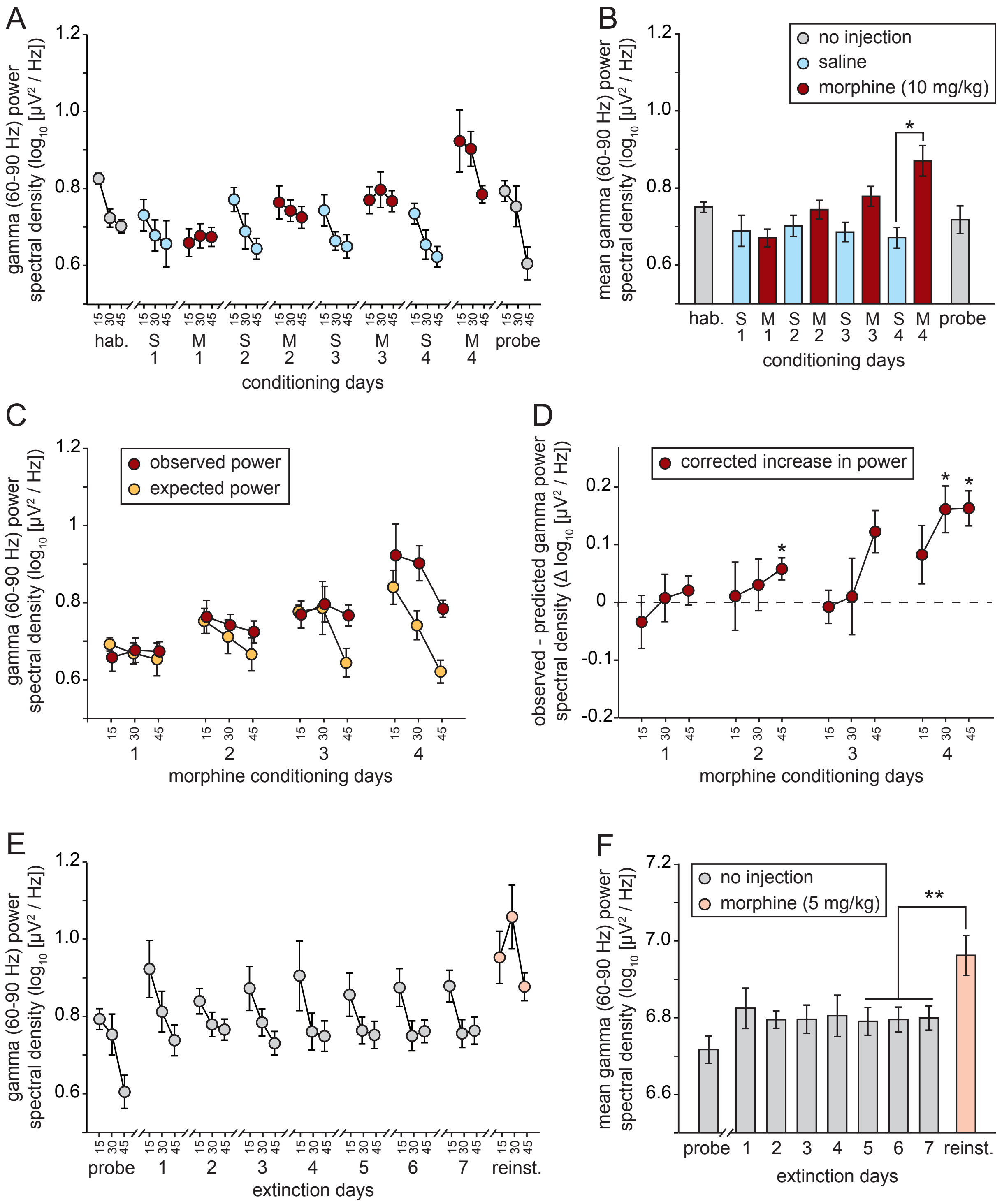

Figure S4. Morphine-induced sensitization of ventral hippocampal high-gamma activity. (A) Gamma power spectral density during habituation (hab.), conditioning, and probe trials, divided into 15-min time bins (S1 = saline day 1; M1 = morphine day 1, etc.). (B) Mean gamma power spectral density during the whole 45 min of each of the trials plotted in A [*$p < 0.05$; paired-sample t-tests with Benjamini-Hochberg correction for multiple comparisons]. (C) Observed power spectral density (divided into 15-min time bins) during morphine conditioning days, versus power predicted based on the positive relationship between locomotor activity and gamma power; see Fig. S2B. (D) Morphine-induced increase in gamma power corrected for locomotor activity [*$p < 0.05$; one-sample t-tests with Benjamini-Hochberg correction for multiple comparisons]. (E) Gamma power spectral density during probe, extinction, and reinstatement (reinst.) trials, divided into 15-min time bins. (F) Mean gamma power spectral density during the whole 45 min of each of the trials plotted in E [**$p < 0.01$; paired-sample t-test].
